# Professional Healthcare Networks for Older Adults Living With Type 2 Diabetes

**DOI:** 10.1093/geroni/igaf122.570

**Published:** 2025-12-31

**Authors:** Catherine Clair, Janiece Taylor, Karin Tobin

**Affiliations:** Johns Hopkins School of Nursing, Baltimore, Maryland, United States; Johns Hopkins University, Baltimore, Maryland, United States; Johns Hopkins Bloomberg School of Public Health, Baltimore, Maryland, United States

## Abstract

Older adults living with Type 2 diabetes often interact with healthcare providers in outpatient and home settings. These healthcare providers represent a professional healthcare network, which can be explored using egocentric social network methods and analysis. We aimed to 1) explore network characteristics in a sample of older adults living with Type 2 diabetes and 2) identify individual- and network-level variables associated with the number of emergency department visits. We conducted a cross-sectional observational study, which included a social network inventory, with community-dwelling older adults (65+ years) living with Type 2 diabetes. We ran a negative binomial regression of emergency department utilization on number of professional alters in the network providing care for five years or more, adjusting for the number of additional chronic conditions, race, driving status, and dual eligibility for Medicare and Medicaid. The final sample included 122 individuals and was 70.5% female (n = 86) and 57.4% non-White (n = 70). The mean age of participants was 73.06 ± 6.12 years. Of the 122 participants, 121 had a professional healthcare network. In adjusted analyses, for each additional professional alter in their network providing care for five years or more, older adults had 0.91 times the incidence rate of emergency department visit (95% CI: 0.80-1.04) when controlling for other covariates of interest. Although this pilot study was not powered for significance, our findings highlight the importance of care continuity and suggest a need for future confirmatory studies to explore the impact of care continuity on the health and well-being of this population.

